# The Paradox of Clean Eating: Neuroactive Dysbiosis and Pesticide Residues in Fruit- and Vegetable-Based Diets

**DOI:** 10.3390/toxics13060504

**Published:** 2025-06-15

**Authors:** Ramona Alina Tomuța, Andrada Florina Moldovan, Loredana Matiș, Lavinia Maris, Timea Claudia Ghitea, Florin Banica

**Affiliations:** 1 Doctoral School of Biological and Biomedical Sciences, University of Oradea, 1 University Street, 410087 Oradea, Romania; yasmine_tomas@yahoo.com; 2 Medicine Department, Faculty of Medicine and Pharmacy, University of Oradea, 1 University Street, 410087 Oradea, Romania; onita.andrada@yahoo.com (A.F.M.); matisloredana@yahoo.com (L.M.); 3 Faculty of Medicine, “Vasile Goldis” Western University of Arad, 94 Revolutiei Blvd., 310130 Arad, Romania; laviniamaris2010@yahoo.com; 4 Pharmacy Department, Faculty of Medicine and Pharmacy, University of Oradea, 1 University Street, 410087 Oradea, Romania; florinbanica1@gmail.com

**Keywords:** pesticide residues, organophosphates, gut microbiome, neuroactive bacteria, chlorpyrifos, dietary exposure, GABA modulation, food safety

## Abstract

(1) Background: Exposure to pesticide residues through food remains a critical issue in public health, especially given their potential cumulative neurotoxic effects. (2) Methods: This study investigated the presence of pesticide residues in commonly consumed vegetables, fruits, and cereals based on official laboratory reports and evaluated the intestinal microbiome profiles of individuals whose diets consisted of over 50% plant-based foods. (3) Results: Analytical results from accredited laboratories in Romania demonstrated that all tested food samples were compliant with European regulations (Regulation (EC) 396/2005), with either undetectable or below-quantification-limit pesticide residues. However, organophosphates such as chlorpyrifos and diazinon were frequently tested, indicating persistent regulatory concern due to their known neurotoxic potential. A parallel analysis of stool samples revealed significant imbalances in neuroactive gut bacteria, including consistently low levels of *Bifidobacterium* and *Lactobacillus* species, and elevated levels of *Oscillibacter* and *Alistipes*, which are implicated in modulating GABA and serotonin pathways. Markers of proinflammatory activity, such as LPS-positive bacteria and histamine producers, were also elevated. (4) Conclusions: These findings suggest that even in diets rich in plant-based foods, microbial dysbiosis with neuroactive relevance can occur, potentially linked to environmental or dietary factors. The study underscores the need for a comprehensive evaluation of food safety and microbiome function as interconnected determinants of neurological health.

## 1. Introduction

Pesticides are a broad class of chemical substances used in agriculture to prevent, control, or eliminate pests that threaten crop productivity. These include insecticides, fungicides, herbicides, and other agents used for plant protection. While they contribute significantly to food security and agricultural efficiency, the widespread and sometimes inappropriate use of pesticides has raised growing concerns about their long-term effects on human health and the environment [1,2,3].

Recent research has demonstrated that even low-dose pesticide exposure may modulate gut microbiota composition, acting as an environmental driver of dysbiosis. Given the role of the gut–brain axis in regulating inflammation, neurotransmission, and mood, understanding how dietary contaminants affect microbial balance has significant implications for neurological and systemic health [4,5].

Human exposure to pesticide residues occurs predominantly through the consumption of plant-based foods but also via contaminated water, inhalation, or dermal contact. Even when residue levels fall below the maximum residue limits (MRLs) established by European regulations, increasing evidence suggests that chronic exposure—particularly to mixtures of substances—may produce cumulative or synergistic effects. Documented health risks include endocrine disruption, neurotoxicity, immune dysregulation, reproductive toxicity, and possible carcinogenicity [6,7].

Organophosphorus pesticides, such as chlorpyrifos and diazinon, are particularly scrutinized due to their neurotoxic potential. Chronic low-level exposure has been associated with cognitive impairments, altered behavior, and an increased risk of neurodegenerative diseases. Proposed mechanisms include oxidative stress, neuroinflammation, the dysregulation of neurotransmitter synthesis (notably acetylcholine and GABA), and blood–brain barrier disruption. Epidemiological studies further associate prenatal and occupational exposure with a higher prevalence of Parkinson’s disease, Alzheimer’s disease, and autism spectrum disorders [8,9,10,11].

Monitoring and controlling pesticide residues in food is a public health priority regulated under Regulation (EC) No. 396/2005. In Romania, such testing is conducted by the network of accredited laboratories within the National Sanitary Veterinary and Food Safety Authority (ANSVSA) [12,13].

While organophosphates such as chlorpyrifos and diazinon have received significant attention for their neurotoxic potential, other pesticide classes—including neonicotinoids (e.g., imidacloprid), pyrethroids (e.g., fenpropathrin), and systemic fungicides (e.g., difenoconazole, fludioxonil)—were also frequently tested in our dataset. These compounds are increasingly associated with subclinical neurotoxic effects, endocrine disruption, and microbiome alterations. Importantly, although residue levels are within legal limits, chronic exposure to mixtures of these agents may lead to additive or synergistic toxicological effects [14]. Experimental models suggest that combined exposure to organophosphates and pyrethroids, for example, can enhance oxidative stress, mitochondrial dysfunction, and neuroinflammation [15,16].

In this context, the present study had a twofold aim: first, to evaluate pesticide residue profiles in fruit, vegetable, and cereal samples from Romania and imported sources, based on official laboratory testing; and second, to assess gut microbiota profiles in individuals consuming diets with more than 50% plant-based foods, with a focus on neuroactive bacterial strains. Through this integrated approach, the study sought to explore potential correlations between dietary exposure, microbial dysbiosis, and neurofunctional health risks.

## 2. Materials and Methods

### 2.1. Data Sources

This study integrated two major datasets. First, we analyzed a series of official analysis bulletins issued by accredited laboratories within the National Sanitary Veterinary and Food Safety Authority (ANSVSA) network in Romania, covering the period 2024–2025. These documents report results from national surveillance and veterinary food safety controls on non-animal food products, primarily vegetables, fruits, and cereals.

Each food sample was analyzed as part of a routine surveillance batch (3–5 subsamples per product), sourced directly from retail outlets and stored under refrigeration (4 °C) prior to transport to the testing facility. Samples were analyzed in their raw, unwashed form to reflect realistic consumer exposure. Pesticide residue analysis followed ISO/IEC 17025-accredited protocols [17].

Second, we included gut microbiota profiles of individuals consuming predominantly plant-based diets, analyzed through a certified stool testing protocol (GanzImmun Diagnostics GmbH, Hans-Böckler-Straße 109, 55128 Mainz, Germany). These results provided insight into microbial composition, diversity, and functional activity, particularly regarding neuroactive and proinflammatory bacterial groups.

### 2.2. Types of Samples Analyzed

Food Products (Pesticide Testing):

- Vegetables: potatoes, cucumbers, tomatoes.
- Fruits: apples.
- Cereals and derivatives: wheat, white flour.
- Other plant-based items: seeds, canned goods.

Human Samples (Microbiome Testing):

Stool samples collected from 50 adults, aged 18–65, who had consumed a diet composed of at least 50% fruits and vegetables for a minimum of 6 months. Each participant consumed at least 1 of these foods daily: cucumbers, tomatoes, mango, potatoes, apple, lemon. All individuals were considered apparently healthy but reported gastrointestinal complaints such as bloating, flatulence, constipation, diarrhea, or postprandial fatigue during the previous 6 months.

Detection Methods Used

Pesticide Residue Testing:

- GC-MS/MS (Gas Chromatography–Tandem Mass Spectrometry): for detection of a broad range of pesticides including organochlorines, organophosphates, pyrethroids, and carbamates.
- LC-MS/MS (Liquid Chromatography–Tandem Mass Spectrometry): for less volatile and systemic pesticide compounds.
- Microscopic methods: used for visual detection of mycotoxins and biological contaminants (e.g., ergot bodies, sclerotia).
- LOD and LOQ determinations: each bulletin reported the detection limit (LOD) and quantification limit (LOQ), and results were annotated accordingly.

Microbiome Analysis:

- Testing was conducted using the GanzImmun stool microbiome profile, including the following analyses:
- Quantitative and qualitative analysis of dominant phyla (Firmicutes, Bacteroidetes, Actinobacteria, etc.).
- Functional subgroup profiling (e.g., butyrate producers, GABA-modulating species, LPS-positive bacteria).
- Shannon diversity index and Firmicutes/Bacteroidetes ratio.
- Detection of neuroactive and proinflammatory species (e.g., *Alistipes* spp., *Oscillibacter* spp., *Bifidobacterium* spp., *Lactobacillus* spp., *Clostridium* spp.).

### 2.3. Interpretation Criteria

Pesticide Results

Results were interpreted according to the following criteria:

- Maximum residue limits (MRLs) as per Regulation (EC) No. 396/2005.
- Regulatory compliance status (compliant/non-compliant).

Pesticide concentration was categorized as follows:

- “Not detectable” (<LOD);
- “<LOQ” (detected but not quantifiable).
- Concentration in mg/kg (if above LOQ).

Microbiome Results

- Evaluated in relation to reference population ranges provided by GanzImmun.
- Key parameters included diversity (Shannon index > 2.7), mucosal protection, proinflammatory activity, and neuroactive potential.

### 2.4. Objective of the Assessment

This study aimed to provide an integrated evaluation of dietary pesticide exposure and gut microbiota integrity in the context of plant-rich diets. The first component characterized the safety profile of widely consumed plant foods in Romania, while the second examined whether microbiota composition in individuals with healthy dietary patterns but persistent digestive symptoms revealed neuroactive or inflammatory imbalances that may suggest subtle toxicological or dietary influences.

### 2.5. Ethical Considerations

This study was conducted in accordance with the principles of the Declaration of Helsinki and the European General Data Protection Regulation (GDPR 2016/679). All participants included in the microbiome analysis provided informed consent prior to sample collection and data usage. Personal data were anonymized and processed solely for scientific purposes. As the microbiome samples were collected as part of routine clinical diagnostics and the study design was observational, no interventional procedures were applied. The study protocol was reviewed and approved by the local ethics committee affiliated with the coordinating institution.

### 2.6. Statistical Analysis

Descriptive statistics were used to summarize the characteristics of both the food samples and microbiome data. Pesticide concentrations were classified according to detection thresholds (LOD, LOQ, MRL) and compliance with EU standards. For microbiota data, relative abundances were compared against reference intervals provided by the testing laboratory (GanzImmun Diagnostics GmbH). The frequency of abnormal bacterial values (either above or below reference) was calculated as counts and percentages across the study population. When appropriate, comparisons between groups were made using non-parametric methods (e.g., Mann–Whitney U test) due to the non-Gaussian distribution of microbial data. Analyses were performed using Microsoft Excel version 4.2 and SPSS version 20.

**Figure 1:**
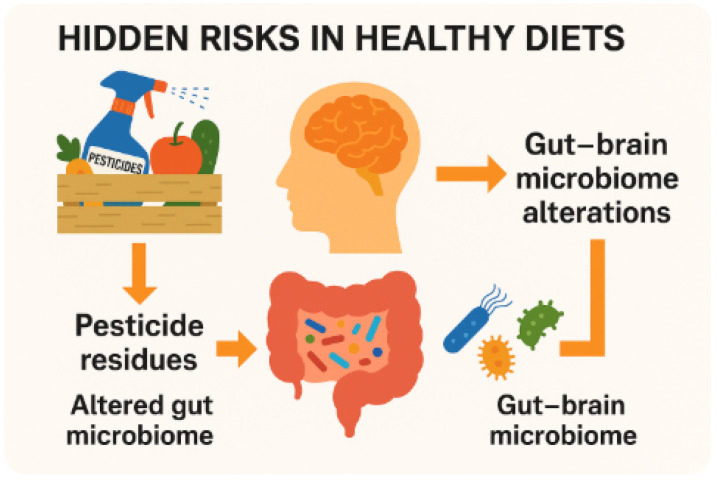
Graphical abstract. Hidden risks in healthy diets: pesticide residues may alter gut microbiota composition, which in turn may affect gut–brain signaling and contribute to neuroactive dysbiosis.

The hidden risks in healthy diets are presented in Figure 1.

## 3. Results

### 3.1. Subsection

Table 1 summarizes the results of pesticide and contaminant analyses for six categories of plant-based food products, including vegetables (cucumbers, tomatoes, potatoes), fruits (apples), and cereals (wheat, white flour). The food items originated from both domestic sources (Romania) and international imports, particularly from Turkey and Poland. Each product was screened for a range of active substances, including organophosphorus compounds such as chlorpyrifos and fosthiazate, pyrethroids like fenpropathrin, neonicotinoids such as imidacloprid, and, in the case of cereals, ergot alkaloids and sclerotia. The number of substances tested varied considerably by product, with more comprehensive testing (up to 100 substances) performed for imported vegetables such as potatoes and cucumbers.

Across all categories, the laboratory reports indicated that the pesticide residues were either below the detection threshold (LOD), below the quantification limit (LOQ), or, in the single case of detected imidacloprid in tomatoes, present in concentrations compliant with the maximum residue levels (MRLs) established by European Regulation (EC) No. 396/2005. No non-compliant samples were found, and all products met current EU food safety standards.

**Figure 2:**
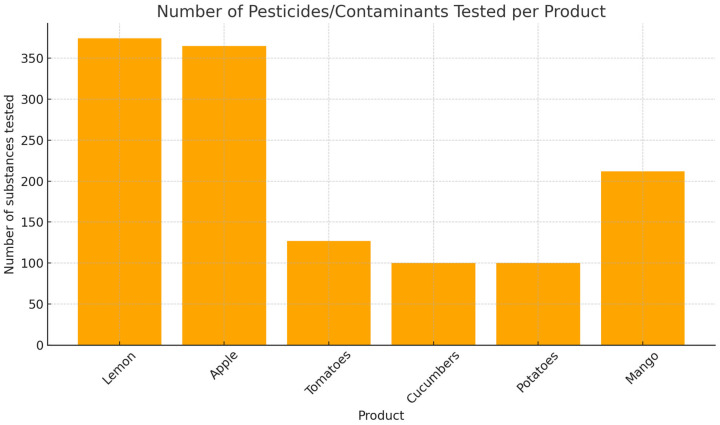
Classification of the six food product categories (tomato, cucumber, lemon, mango, potato, apple) according to pesticide residue detection and compliance with EU maximum residue levels (MRLs) per product sample (2024–2025).

Although no MRL exceedances were reported, the high frequency of screening for substances with well-documented health risks—including endocrine disruptors and neurotoxic compounds—emphasizes the importance of continued monitoring and suggests the need for further investigation into cumulative dietary exposure, particularly in the context of long-term consumption of treated plant-based foods. Figure 2 classifies all food products analyzed in this study (*n* = 6 categories), including tomatoes, cucumbers, apples, potatoes, lemon, and mango, based on the presence and regulatory status of detected pesticide residues.

This chart emphasizes the significantly higher number of pesticide compounds screened in lemon and apple samples, suggesting increased regulatory scrutiny or known risk of contamination for these products.

### 3.2. Distribution of Results Regarding the Presence of Pesticides

**Figure 3:**
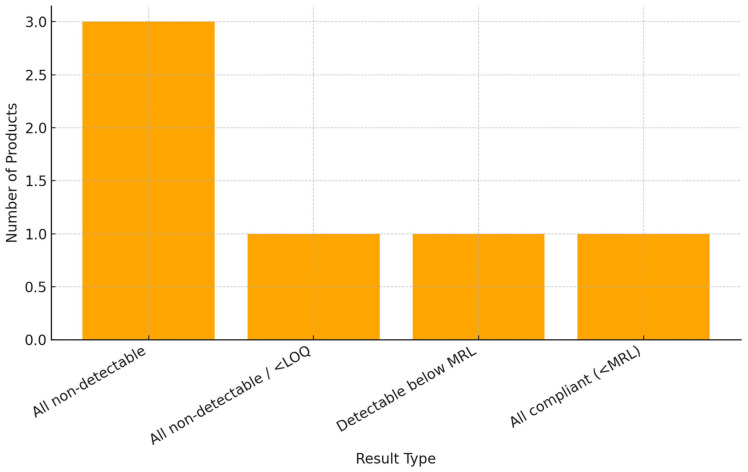
Classification of food samples based on pesticide residue detection and compliance with MRLs.

Figure 3 illustrates that most products (three out of six) showed no detectable pesticide residues. In one product each, residues were found but either below the quantification limit (LOQ), detectable but below the MRL, or simply all compliant under MRL thresholds.

### 3.3. Gut Microbiome Profiles in Individuals with a Plant-Rich Diet

Five individual stool microbiome reports were analyzed, all obtained through GanzImmun Diagnostics GmbH. Participants had reported consuming diets composed of over 50% fruits and vegetables over the previous six months. Although all individuals were considered apparently healthy, they reported persistent gastrointestinal symptoms, including bloating, belching, constipation, diarrhea, or postprandial fatigue. The aim was to detect patterns of microbial dysbiosis with potential neurofunctional relevance.

#### 3.3.1. Neuroactive Dysbiosis—Frequently Altered Bacteria

The microbiome analysis revealed consistent alterations in neuroactive bacterial taxa. Key findings are summarized in the Table 2 below.

These findings suggest the depletion of GABA-producing and barrier-protecting bacteria, alongside an increase in valeric acid-producing species, which may alter neurochemical balance along the gut–brain axis.

#### 3.3.2. Associated Functional Alterations

Additional microbial patterns indicative of systemic or neuroinflammatory potential were also observed:

- LPS-positive (endotoxin-producing) bacteria were elevated in three out of five individuals, suggesting increased systemic inflammatory tone.
- *Faecalibacterium prausnitzii*, a known anti-inflammatory commensal, was present at subnormal levels in four out of five samples.
- Histamine-producing bacteria, such as *Clostridium* spp., were increased in the majority of cases, indicating a higher risk for neuroinflammatory processes.

These alterations occurred despite a predominantly plant-based diet, highlighting that microbiome health depends not only on food categories but also on microbial diversity, fermentable fiber intake, and the presence of beneficial bacterial strains. The observed dysbiotic patterns are consistent with increased risks for the following:

- nervous system hyperexcitability;
- sleep and mood disorders;
- chronic neuroinflammation and potentially neurodegenerative processes.

**Figure 4:**
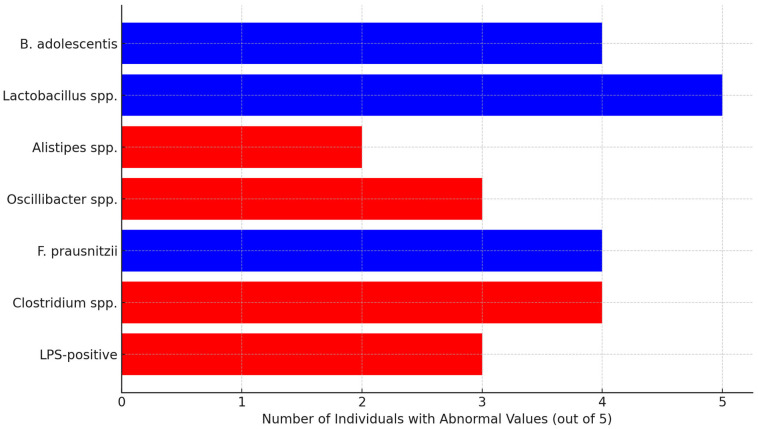
Alterations in neuroactive and inflammatory bacteria in the gut microbiome. Blue bars represent reduced neuroactive/protective bacteria; red bars represent elevated inflammatory or neuroactive-risk species.

Figure 4 illustrates the frequency of abnormal values observed in key neuroactive and inflammatory bacterial taxa across five individuals with plant-rich diets. Notably, *Lactobacillus* spp. were absent in all participants, and *Bifidobacterium adolescentis* was below reference levels in four out of five cases—both critical for GABA production and intestinal barrier integrity. Similarly, *Faecalibacterium prausnitzii*, an anti-inflammatory butyrate producer, was reduced in 80% of cases.

Conversely, increased levels of *Clostridium* spp. and LPS-positive bacteria (e.g., *Klebsiella*, *Escherichia*) were found in three or more subjects, indicating a proinflammatory microbial environment. Additionally, *Oscillibacter* spp. and *Alistipes* spp.—bacteria involved in valeric acid and serotonin metabolism—were elevated in several samples, suggesting shifts in neuroactive signaling along the gut–brain axis.

Together, these patterns support the presence of a functionally significant dysbiosis, potentially linked to neuroinflammatory states, even in individuals consuming diets high in vegetables and fruits.

## 4. Discussion

The results obtained from the analysis of official pesticide surveillance bulletins and gut microbiota profiles in individuals consuming predominantly plant-based diets (>50% vegetables and fruits) provide a nuanced perspective on the interplay between dietary exposure, chemical contaminants, and neuropsychiatric health. This dual approach highlights not only the importance of regulatory food safety measures but also the emerging relevance of gut microbiota as a mediator of environmental and dietary influences on brain function.

### 4.1. Pesticides and Neurotoxicity

Although all food samples in this study complied with current European legislation on pesticide residues, specifically Regulation (EC) No. 396/2005, there remains considerable concern regarding the long-term effects of chronic low-dose exposure. Organophosphorus compounds, such as chlorpyrifos and diazinon, exert their neurotoxicity through inhibition of acetylcholinesterase, leading to the accumulation of acetylcholine at neuronal synapses and subsequent disruption of cholinergic signaling [9,18]. This mechanism underpins a growing body of evidence linking chronic exposure to these pesticides with cognitive decline, mood disturbances, and an increased risk of neurodegenerative diseases such as Parkinson’s disease, Alzheimer’s disease, and autism spectrum disorders [19]. The subtlety of their effects at low concentrations makes them particularly insidious, as they may act cumulatively and synergistically with other environmental factors over time [20].

### 4.2. Risks Associated with MRL Exceedances

Although no pesticide maximum residue level (MRL) exceedances were observed in the present dataset, European Food Safety Authority (EFSA) monitoring programs consistently report that 2–5% of food samples annually exceed these thresholds [21,22]. Such cases are more frequently associated with imported products or those subjected to intensive pre-harvest treatments. Repeated consumption of such foods could pose significant risks, particularly for vulnerable populations such as children, pregnant women, and individuals with pre-existing hepatic or renal conditions [23]. Beyond acute toxic effects, excessive pesticide intake has been implicated in endocrine disruption, immune dysfunction, and neurobehavioral alterations. Organophosphates and carbamates, in particular, are associated with persistent alterations in neuronal excitability and glial activation [10,24]. These observations underscore the need for not only continuous monitoring but also swift response systems for the detection and removal of contaminated food batches.

### 4.3. Microbiota and the Gut–Brain Axis

The microbiota analysis in individuals with a high intake of fruits and vegetables revealed consistent imbalances in bacterial taxa with known neuroactive functions [25]. Key GABA-producing species, such as *Bifidobacterium adolescentis* and strains of *Lactobacillus* (e.g., *L. brevis*, *L. plantarum*), were undetectable or markedly reduced in the majority of samples. In contrast, species associated with valeric acid and serotonin metabolism—*Alistipes* spp. and *Oscillibacter* spp.—were elevated in several individuals. These shifts suggest a dysregulated microbiota with potential consequences for neurotransmitter synthesis and receptor signaling. Notably, increased levels of lipopolysaccharide (LPS)-producing bacteria (e.g., *Escherichia coli*, *Klebsiella* spp.) were observed in three of five cases, indicating a state of low-grade intestinal inflammation that may affect systemic and neuroinflammatory pathways. These findings align with the concept of the gut–brain axis, wherein microbial-derived metabolites directly influence mood, cognition, and immune regulation [26,27].

### 4.4. Dysbiosis Despite a Plant-Based Diet

Paradoxically, despite adherence to a predominantly plant-based diet, which is often associated with positive health outcomes, the participants exhibited microbiota imbalances of functional relevance [28,29]. This highlights the complexity of gut ecology, where factors such as insufficient intake of fermentable fibers (e.g., inulin, resistant starch, pectin), environmental stressors, prior antibiotic use, and residual foodborne contaminants (e.g., pesticides) may disrupt microbial homeostasis [30,31,32]. The absence of protective species and overgrowth of potentially neuroactive or inflammatory strains may contribute to the persistence of gastrointestinal symptoms and subtle alterations in neurobehavioral states, even in the absence of overt disease.

### 4.5. Clinical and Preventive Implications

Our findings suggest that evaluating gut health should extend beyond assessing microbial diversity to include functional profiling of key bacterial groups. Moreover, chronic dietary exposure to pesticide residues—even within legal safety limits—may contribute to cumulative alterations in microbiota composition and gut–brain communication [33]. Plant-based diets, while beneficial, should be carefully structured to ensure the inclusion of prebiotic fibers (e.g., fructooligosaccharides, galactooligosaccharides, pectin) and potentially supported by the use of targeted probiotic strains known to restore GABAergic and anti-inflammatory functions [34,35,36,37,38]. From a public health perspective, integrated strategies combining food safety regulation, dietary education, and microbiome monitoring may offer the most comprehensive protection against chronic low-grade neurotoxicity and inflammation [39,40,41].

### 4.6. Justification for Study Design and Population

The selection of participants consuming diets composed of more than 50% plant-based foods was guided by nutritional recommendations such as Harvard University’s Healthy Eating Plate, which advocates for a high intake of fruits and vegetables. This criterion was chosen to reflect a general healthy eating pattern without focusing exclusively on restrictive dietary groups such as vegans, vegetarians, or ketogenic dieters. While the ≥50% threshold is not a strict boundary, it served as a consistent inclusion parameter that allowed for the exploration of paradoxical gastrointestinal symptoms despite a seemingly healthy diet. Failure to meet this threshold would not invalidate microbiome findings, but it would shift the focus away from the dietary pattern under investigation.

### 4.7. Integration of Databases and Justification

The two main components of this study—pesticide residue profiles and gut microbiome data—were derived from the same cohort of individuals. The pesticide data were obtained from regulatory bulletins of food products commonly consumed by participants, while the microbiota analysis was performed directly on stool samples from these individuals. The rationale for integrating these datasets lies in assessing the interplay between legally compliant dietary exposures and real-time microbial alterations. Though retrospective in dietary recall and symptom assessment, the microbiome data reflect a cross-sectional snapshot of current gut health.

### 4.8. On the Absence of a Control Group

This exploratory study did not include a control group of asymptomatic individuals, as all participants were selected from a clinical nutrition office due to persistent gastrointestinal complaints despite adherence to a plant-rich diet. This paradoxical presentation prompted the investigation. While the inclusion of a control group would strengthen future studies, the current analysis focused on symptomatically affected individuals. A follow-up longitudinal study is currently underway to evaluate whether symptom onset correlates with the duration of dietary exposure or the quality of plant-based foods consumed. Until such data are available, the current study provides a hypothesis-generating foundation that supports future causal investigations.

### 4.9. Limitations

This study presented an integrative analysis of foodborne pesticide residues and gut microbiota profiles in individuals adhering to predominantly plant-based diets. However, several limitations must be acknowledged. First, while the analyzed food samples represent commonly consumed fruits and vegetables in the region, we did not perform residue testing on the exact food items ingested by each participant. Nonetheless, each participant reported daily consumption of at least one of the analyzed products (cucumbers, tomatoes, mango, potatoes, apple, or lemon), supporting the ecological validity of the exposure assessment. Second, the observational design precluded causal inference between dietary pesticide exposure and microbiota alterations. Factors such as medication use, stress, comorbid conditions, and inter-individual dietary variability may have contributed to the observed microbial imbalances. Additionally, the microbiome analysis was performed on a relatively small sample size (*n* = 5 detailed reports), limiting the generalizability of findings. Future studies should include direct matching of dietary intake with food residue analysis and a larger, more diverse participant cohort to more robustly characterize exposure–response relationships.

### 4.10. Future Research Directions

Our findings raise important questions about the link between chronic low-dose pesticide exposure and functional changes in the gut microbiome, especially in populations adhering to health-conscious dietary patterns. Future studies should integrate personalized dietary tracking with direct residue analysis of consumed food items, alongside larger-scale microbiome profiling. Intervention studies using prebiotics or probiotic strains targeting GABAergic and anti-inflammatory pathways may help assess the reversibility of pesticide-associated dysbiosis. Furthermore, multi-omics approaches—including metabolomics and transcriptomics—may provide deeper insights into host–microbe–xenobiotic interactions.

## 5. Conclusions

This study provides an integrated assessment of dietary pesticide exposure and intestinal microbiota composition in individuals adhering to predominantly plant-based diets. The results offer important insights into the hidden risks that may persist even within the framework of “healthy” dietary habits.

Pesticide residue analyses revealed that all tested food samples were compliant with current European legislation, with residues either undetectable, below the quantification limit, or within acceptable maximum residue levels (MRLs). However, the frequent detection of compounds with known neurotoxic potential, such as organophosphates, underscores the importance of continued surveillance and risk assessment, particularly in the context of chronic low-dose exposure and cumulative effects.

The parallel analysis of gut microbiota profiles in individuals consuming >50% fruits and vegetables revealed a pattern of neuroactive dysbiosis, characterized by the depletion of beneficial GABA-producing and anti-inflammatory bacteria, and the overgrowth of species involved in inflammatory and neurochemical disruption. These alterations were observed despite the absence of traditional dietary risk factors and were accompanied by common gastrointestinal complaints, suggesting an underlying functional imbalance.

Together, these findings highlight the need for a more holistic approach to food safety and health promotion—one that considers not only chemical compliance but also microbial functionality. Future interventions should focus on increasing dietary prebiotic diversity, monitoring microbiome health, and reducing long-term exposure to pesticide residues through sustainable agricultural practices and informed consumer choices.

Practical Applications

These findings support the growing need for integrative food safety assessments that consider not only legal compliance with residue limits but also functional impacts on gut health. The results may inform consumer education, dietary guidance, and agricultural practices aimed at minimizing chronic chemical exposure while supporting microbiome integrity.

## Figures and Tables

**Table 1:** Summary of pesticide and contaminant residue analysis in plant-based food products (2024–2025).

Product	Country of Origin	Substances Tested (Example)	No. of Substances Tested	Results	According to EU Legislation
Cucumbers	Turkey	Fenpyroximate, fosthiazate, chlorpyrifos	80	All below LOQ	Yes
Tomatoes	Romania	Imidacloprid	1	Detected imidacloprid but below MRL	Yes
Mango	Romania	Chlorpyrifos, fenpropathrin	2	All below LOD	Yes
Potatoes	Poland	Vinclozolin	100	All compliant with Reg. 396/2005	Yes
Apple	Romania	Fludioxonil, propoxur	4	All compliant with Reg. 396/2005	Yes
Lemon	Romania	Pyridaben	1	All compliant with Reg. 396/2005	Yes

**Table 2:** Alterations in neuroactive gut bacteria among individuals with a predominantly plant-based diet.

Neuroactive Bacteria	Primary Role	Below Normal?
*Bifidobacterium adolescentis*	GABA production	Yes (in 4 out of 5 cases)
*Lactobacillus brevis*	GABA production, intestinal barrier integrity	Absent in all 5 cases
*Alistipes* spp.	Serotonin metabolism, valeric acid production	Elevated (>6.7%) in 2 cases
*Oscillibacter* spp.	Valeric acid producer, modulator of GABA pathways	Elevated (>0.3%) in 3 cases

## Data Availability

All the data processed in this article are part of the research for a doctoral thesis, being archived in the aesthetic medical office where the interventions were performed.
